# Comparative analysis of vascular bulldog clamps used in laparoscopic liver resection

**DOI:** 10.1097/MD.0000000000026074

**Published:** 2021-06-11

**Authors:** Liang He, Weixiang Li, Dachen Zhou, Lei Wang, Hui Hou, Xiaoping Geng

**Affiliations:** Department of Hepatobiliary Surgery, The Second Hospital of Anhui Medical University, Hefei, Anhui, PR China.

**Keywords:** bulldog, laparoscopic hepatectomy, tourniquet, vascular occlusion

## Abstract

To compare the clinical effect of Bulldog clamps with traditional Pringle for vascular occlusion during laparoscopic hepatectomy.

One hundred ten patients were retrospectively investigated in this research from December 2014 to January 2019 in the second hospital of Anhui Medical University, who underwent laparoscopic liver resection using Bulldog (modified group, n = 54) and cotton tourniquet (traditional group, n = 56) for blocking the liver inflow-blood. Intraoperative blood loss, duration of the operation time, clamping time, postoperative outcomes were analyzed.

All the operations were accomplished successfully without conversion to laparotomy, perioperative period clinical date was calculated. Intraoperative operative time, blood loss and resection sections had no statistical significance, but the clamping time (36.2 ± 5.6 vs 277.3 ± 88.4 s, *P* < .001) was significantly shorter in the bulldog group. Albumin, alanine aminotransferase, aspartate aminotransferase and serum total bilirubin had no statistical differences in postoperative day (POD) 1and 3, but POD 5 alanine aminotransferase (71.0 ± 46.8vs 105.8 ± 61.7IU/L *P* = .018) and aspartate aminotransferase (72.8 ± 39.7 vs 100.2 ± 16.7 IU/L *P* = .028). The postoperative hospital stays (7.02 ± 1.56 vs 8.50 ± 2.35 days *P* = .026) in bulldog group were lower than cotton group and differences had statistical significance. The C-reactive protein levels were significantly higher in the traditional group than in the modified group on POD 3 (46.3 ± 19.2 vs 57.7 ± 23.9 mg/L *P* = .019), and POD5 (13.3 ± 4.2 vs 17.5 ± 7.3 mg/L *P* = .001). There were 8 postoperative complications occurred in cotton group, while there was 5 in Bulldog group, all patients with complications were discharged after adequate drainage and symptomatic treatment.

Bulldog is an effectively performed approach for vascular occlusion during laparoscopic hepatectomy than traditional Pringle maneuver.

## 1 Introduction

Since the Louisville statement in 2009, the use of laparoscopic liver resection (LLR) has gradually increased in recent years.^[1,2]^ LLR is used for benign tumors located on the lateral surface with solitary lesions of 5 cm or less located in liver segments 2 to 6, which can be resected more easily than segments VII and VIII.^[3–7]^ With the development of surgical instruments and the accumulation of surgeon experience, LLR has been expanded for hepatocellular carcinoma (HCC) treatment, major liver resection, complex hepatectomy and donor hepatectomy by experienced staff.^[8]^ In 2016,^[9]^ the first Asia Pacific consensus meeting on HCC was held in conjunction with the 7th Asia-Pacific Primary Liver Cancer Expert Meeting in Hong Kong to declare that the advantage of laparoscopic hepatectomy is less intraoperative bleeding and faster postoperative liver function recovery if the procedure is performed by experienced surgeons. Laparoscopic major hepatectomy for HCC remains a challenging technique and should only be performed by experienced surgeons. However, postoperative mortality, morbidity and liver function recovery are associated with major blood loss,^[10]^ which is always the main cause of conversion to laparotomy^[11]^ and remains a challenge for surgeons.^[12,13]^ Pringle^[14]^ first described the method to stop hepatic hemorrhage with compression of the porta hepatis, and this procedure is currently widely used in laparoscopic fields. Here, we described a newly modified Pringle maneuver using Bulldog clamps to block the vasculature during LLR and compared its effects with the traditional Pringle maneuver.

## 2 Methods

### 2.1 Patient population

From December 2014 to January 2019, 200 patients underwent LLR in our department at the 2nd Hospital of Anhui Medical University, and 110 patients underwent LLR with vascular occlusion. There were 54 patients in the Bulldog group and 56 patients in the traditional group. A total of 38 female and 72 male participants were included in this study, with a mean age of 56.1 ± 10.9 and 60.0 ± 8.8 years, respectively. The clinical characteristics of the patients are summarized in Table 1.

**Table 1:** Patient basis characteristics (mean ± SD).

Patient group characteristic	Bulldog (n = 54)	Cotton (n = 56)	*T*(*χ*^*2*^)	*P value*
Age(yr)	56.1 ± 10.9	60.0 ± 8.8	2.683	.104
Gender(male/female)^a^	39/15	33/23	2.149	.143
HBV	42	47	0.673	.412
HCV	3	2	0.249	.617
AFP			0.015	.903
≥7(ng/ml)	38	40		
<7(ng/ml)	16	16		
ALT	44.2 ± 33.1	40.2 ± 38.7	0.585	.560
AST	40.2 ± 23.4	39.4 ± 27.4	0.410	.523
ALB	38.7 ± 7.9	38.5 ± 4.6	0.143	.887
TBIL(umol/l)	16.5 ± 9.5	16.9 ± 8.1	0.01	.972
CRP(mg/L)	4.1 ± 2.2	4.5 ± 2.7	0.90	.370
Liver cirrhosis	44	46	0.008	.928
Child-Pugh(A/B)	52/2	55/1	0.381	.537
Diagnoses			0.388	.824
Hepatic cancer	39	42		
hepatic benign tumor	10	8		
Calculusof intrahepatic duct	5	6		

AFP = alpha fetoprotein, ALB = albumin, ALT = alanine aminotransferase, AST=aspartate aminotransferase, CRP C-reactive protein, HBV = hepatitis B virus, HCV = hepatitis C virus, SD = standard deviation, TBIL = serum total bilirubin.

### 2.2 Inclusion and exclusion criteria

This study was approved by the ethics committee of The Second Hospital of Anhui Medical University. Each participant in the study provided written informed consent. The inclusion criteria were as follows:

1. all the participants corresponded with the application of LLR, according to the Louisville Statement,^[2,8]^ and underwent laparoscopic liver resection;
2. Bulldog clamps and cotton tape were applied during the operation;
3. the tumor did not invade the main vessels; and
4. the Pringle maneuver clamping time was consistently 15 minutes, and the right Glisson pedicle was clamped only once when only right hepatectomy was performed.

LLR was not limited to the disease but included benign tumors, malignant tumors, calculus disease and others (Table 1). Cases that were not in agreement with the inclusion criteria were excluded.

### 2.3 Surgical procedure

**Figure 1:**
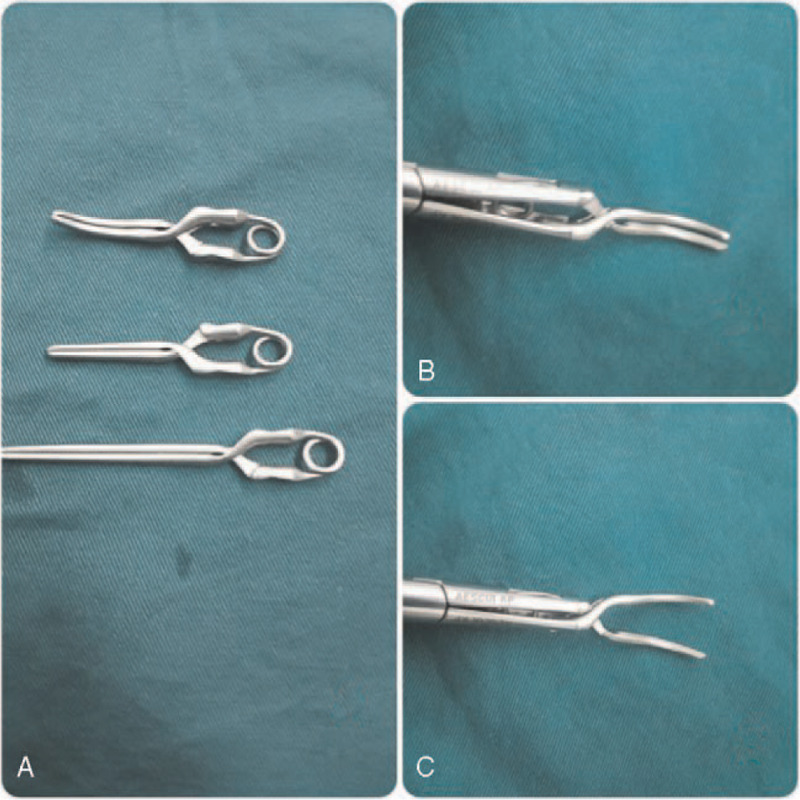
Bulldog A: the different lengths of Bulldog, the longer was used to pringle maneuver, the other were used to the right Glission pedicles, the unique curved design fits better with the baseline than ordinary straight clamp, further ensuring complete vascular occlusion. B: the matched forceps, nonworking status. C: working status.

**Figure 2:**
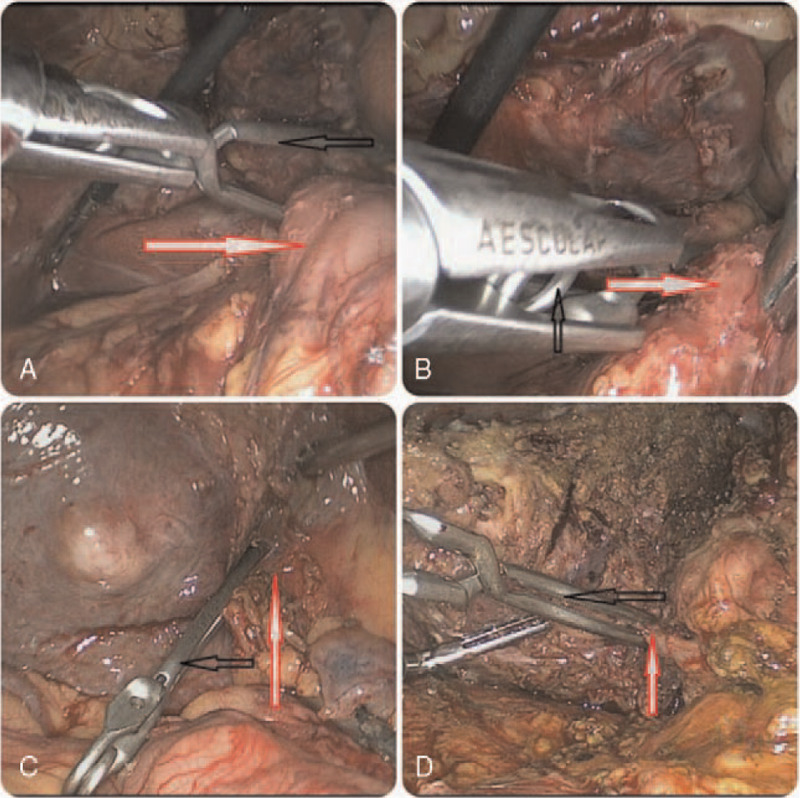
The Bulldog in intraoperative pictures. A and B: red arrows represent hepatoduodenal ligament, C and D: red arrows represent the anatomized right Glission pedicle. A: The forceps hold the Bulldog into the abdominal cavity and start to infibulate the hepatoduodenal ligament, it is easy to operate. B: the bulldog can securely clap the hepatoduodenal ligament. C and D: Bulldog was clamped the right Glission pedicles. All the black arrows represent bulldog.

**Figure 3:**
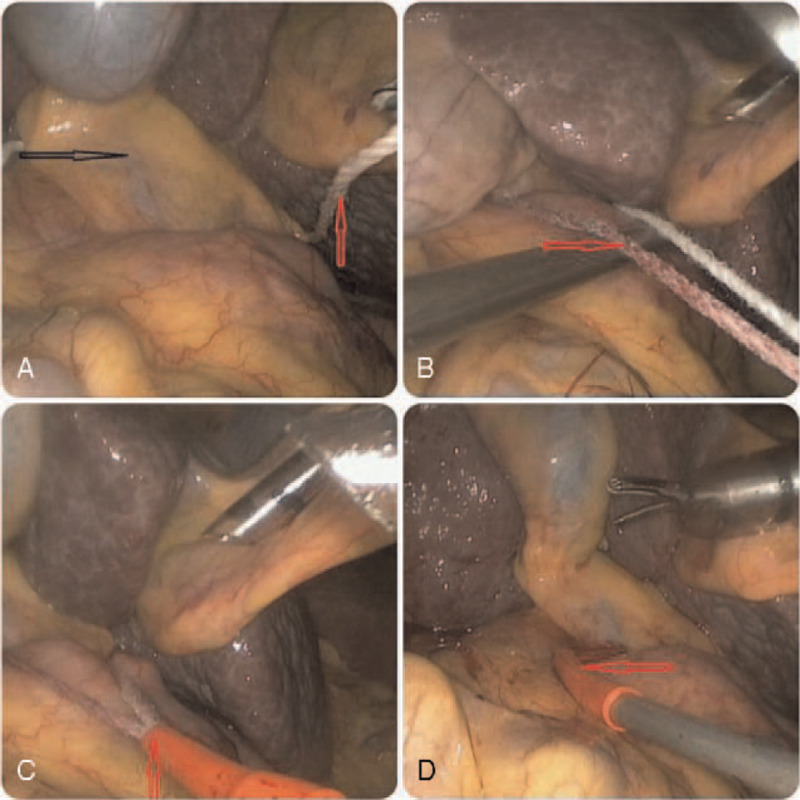
The cotton in intraoperative pictures. This picture shows the process of clamping with cotton during laparoscopic hepatectomy through Extracorporeal methods. A: the cotton was passed behind the hepatoduodenal ligament, B: fasten the cotton .C and D: the tube was used to encircle the hepatoduodenal ligament, and kept the end of the tape out, and fasten by pulling tape through the tube.

General anesthesia was performed routinely. The patients were placed in the supine position, and a 10-mm trocar was placed 2 cm to the right of the umbilicus under direct vision. Intraabdominal pressure was established and maintained at approximately 14 mm Hg, and central venous pressure was set below 5 cmH_2_O.^[15]^ The remaining 4 trocars were placed based on the tumor position. Intraoperative laparoscopic ultrasound was used as a requirement. Liver parenchymal transection was performed using a combination of a harmonic scalpel and bipolar forceps. For patients who underwent the Pringle maneuver, the cotton tape and Bulldog clamping methods were applied. In the Bulldog (Fig. 1) group, we routinely explored the abdominal cavity, dissociated the mucosal tissue around the hepatoduodenal ligament, and exposed the hepatic portal vein as well as the right Glisson pedicle. A Bulldog clamp was delivered into the abdominal cavity with the matched forceps through the 12-mm trocar to block the hepatoduodenal ligament or the right Glisson pedicle (Fig. 2) to block the hepatic inflow. In the cotton clamping group, we needed another 5-mm port trocar, which was positioned in the proper place to ensure that the hepatoduodenal ligament was encircled. Forceps were passed through the hepatic pedicle, and 80-cm cotton tape was placed around the pedicle. Then, the ends of the cotton tape were pulled out through the 5-mm trocar port, and a tube was pushed inside the abdominal cavity close to the hepatic pedicle and was fastened by pulling the cotton tape through the tube^[16]^ (Fig. 3).

### 2.4 Outcomes

All the patients underwent the operation by 1 team and received the same postoperative care. The postoperative complications were recorded and analyzed. C-reactive protein (CRP) and liver function markers, including alanine aminotransferase (ALT), Aspartate aminotransferase (AST), Serum total bilirubin (TBIL)and albumin (ALB), were checked on the first, third and fifth days after liver resection as well as throughout the postoperative duration.

### 2.5 Statistical methods

Data were analyzed with SPSS 17.0 (IBM Corporation, NY). Continuous variables were described as the mean ± standard deviation (SD) and subjected to Student *t* test. The *χ*^*2*^ test (with continuity corrected *χ*^*2*^ if the expected count was < 5) or Fisher exact test was appropriate for categorical data. A *P* value of <.05 was set as statistically significant.

## 3 Results

### 3.1 Clinicopathologic characteristics

The baseline clinical characteristics of the patients are shown in Table 1. A total of 110 patients underwent laparoscopic liver resection with vascular occlusion. The diagnoses of patients with vascular occlusion in laparoscopic liver resection with the use of Bulldog clamps (n = 54) and cotton tape (n = 56) included hepatic cancer combined with liver cirrhosis, hepatic hemangioma and calculus of the intrahepatic duct. We blocked the right Glisson pedicle in right hepatectomy patients, and the remaining patients were blocked with the Pringle maneuver. The patients in the 2 groups were matched for age, sex, body mass index, hepatitis B virus, liver cirrhosis, resection side and diagnosis, as shown in Table 1. All the patients received similar preoperative assessments and postoperative management.

### 3.2 Intraoperative clinical outcomes

The intraoperative clinical outcomes are summarized in Table 2. The number of patients in the Bulldog group in whom the hepatic vessels were blocked was 126 and in the cotton, group was 156. No significant differences were observed in the type of liver resection, duration of surgery or amount of blood loss, but the clamping time was significantly shorter in the Bulldog group than in the cotton group (36.2 ± 5.6 vs 277.3 ± 88.4 s, *P* < .001).

**Table 2:** Intraoperative detail patients(mean ± SD).

	bulldog(54)	cotton(56)	*T*(*χ*^*2*^)	*P* value
Type of resection, n			7.168	.127
Segment II+III	2	3	0.173	.677
Segment II+III+IV	10	18	2.689	.101
Segment V+VIII	13	12	0.110	.741
Segment VI+VII	18	17	0.112	.738
Segment V, VI, VII, VIII	11	6	1.962	.161
Intraoperative blood loss (ml)	371.8 ± 216.2	411.3 ± 216	0.958	.340
Left segment(ml)	179.2 ± 39.6	196.9 ± 73.7	0.768	.448
Right segment(mil)	430.4 ± 209.5	532.8 ± 178.9	2.279	.025
Patients transfused	4 (7.4%)	8 (14.2%)	1.338	.247
Clamping time (s)	36.2 ± 5.6	277.3 ± 88.4	1.760	.000
Operation time (min)	216.9 ± 68.2	236.4 ± 71.2	1.472	.144
Left segment(min)	161.3.4 ± 68.9	172.2 ± 32.9	0.616	.542
Right segment(min)	241.7 ± 54.7	279.7 ± 51.9	3.114	.003
Liver resection time (min)	85.1 ± 23.6	97.5 ± 21.3	1.056	.306
The blocking numbers^#^	126	156	6.614	.010
Left segment(n)	20	45		
Right segment(n)	106	111		

SD = standard deviation. a: clamping time represent the overall time consuming by clamping the tourniquet which obtained from surgery video, ^#^: the summary times of blocking, Segment V, VI, VII, VIII: including V, VI, VII, VIII and right hepatectomy.

### 3.3 Postoperative clinical outcomes

The liver function on postoperative day (POD) 1, 3, and 5 as reflected by the postoperative changes in ALT, AST, TBIL, and PT are shown in Table 3. The mean ALB, ALT, AST, and TBIL values on POD1 were not significantly different from those on POD3, but the ALT (71.0 ± 46.8 vs 105.8 ± 61.7 IU/L *P* = .018) and AST (72.8 ± 39.7 vs 100.2 ± 16.7 IU/L, *P* = .028) values were significantly lower on POD1 than on POD5. The postoperative hospital duration (7.02 ± 1.56 vs 8.50 ± 2.35 days, *P* = .026) in the Bulldog group was lower than that in the cotton group, and the differences were statistically significant. The CRP levels were significantly higher in the traditional group than in the modified group on POD3 (46.3 ± 19.2 vs 57.7 ± 23.9 mg/L *P* = .019) and POD5 (13.3 ± 4.2 vs 17.5 ± 7.3 mg/L *P* = .001). All of the manipulations were performed easily and quickly using Bulldog clamps. Eight postoperative complications occurred in the cotton group, while 5 complications occurred in the Bulldog group, which disappeared after drainage and anti-infection treatment for 5 to 9 days.

**Table 3:** Postoperative clinical course (mean ± SD).

	Bulldog(n = 54)	Cotton(n = 56)	*T*	*P* value
PMT	0 (0)	0 (0)		
PMB	5 (9.26%)	8 (14.3%)		
Hemorrhage	0 (0)	0 (0)		
Biliary fistula	1 (1.85%)	2 (3.57%)		
Celiac infection	1 (1.85%)	1 (1.79%)		
Peritoneal effusion	1 (1.85%)	1 (1.79%)		
Pleural effusion	0 (0)	1 (1.79%)		
Pulmonary infection	1 (1.85%)	1 (1.79%)		
Venous thrombosis	1 (1.85%)	2 (3.57%)		
POD1				
ALB	30.9 ± 4.91	31.5 ± 4.53	0.156	.693
ALT(IU/L)	346.9 ± 267.2	387.8 ± 276.0	1.089	.299
AST(IU/L)	375.7.9 ± 284.1	413.6 ± 257.8	0.001	.976
TBIL(umol/l)	27.1 ± 14.25	28.2 ± 13.3	0.109	.742
CRP(mg/l)	73.7 ± 19.8	75.9 ± 27.5	0.564	.454
POD3				
ALB	34.5 ± 4.8	34.0 ± 3.5	8.269	.445
ALT(IU/L)	192.2 ± 109.4	233.2 ± 146.6	3.149	.079
AST(IU/L)	182.7 ± 138.1	230.9 ± 177.8	3.313	.072
TBIL(umol/l)	23.5 ± 10.9	27.2 ± 11.4	0.936	.336
CRP(mg/l)	46.3 ± 19.2	57.7 ± 23.9	0.811	.019
POD5				
ALB	36.1 ± 4.74	35.6 ± 3.87	1.167	.282
ALT(IU/L)	71.0 ± 46.8	105.8 ± 61.7	5.787	.018
AST(IU/L)	72.8 ± 39.7	100.2 ± 16.7	4.979	.028
TBIL(umol/l)	17.43 ± 8.03	20.9 ± 9.2	0.226	.636
CRP(mg/l)	13.3 ± 4.2	17.5 ± 7.3	3.579	.001
PHS^#^(d)	7.02 ± 1.56	8.5 ± 2.35	5.116	.026

ALB = albumin, ALT = alanine aminotransferase, AST = aspartate aminotransferase, CRP = C-reactive protein, NS = no specific, PHS = postoperative hospital stays, PMB = postoperative mortality, PMT = postoperative mortality, POD = postoperative day, SD = standard deviation, TBIL = serum total bilirubin.

## 4 Discussion

With the innovations of laparoscopic techniques and specialized equipment, laparoscopic liver resection has become the dominant surgical resection approach.^[17]^ In December 2014, laparoscopic hepatectomy was first carried out in our department, and the extracorporeal^[16,18,19]^ Pringle maneuver has been applied in most laparoscopic liver resections in which the blocking of hepatic inflow is needed; cotton tape is frequently applied.^[20]^ Additionally, there have been many novel devices, and Shin-ichiro^[21]^ recommended the smooth and effective features of a biliary scope for the Pringle maneuver in laparoscopic hepatectomy in 2007. The next year, Akihiro^[22]^ investigated 32 consecutive patients with the Endo Retract Maxi for use in the Pringle maneuver during laparoscopic hepatectomy, and Dua MM used umbilical tape^[23]^ in 2015. We used to block hepatic inflow via the extracorporeal Pringle maneuver method with the use of cotton tape^[5]^ due to its validity, softness and safety for vessel protection, but it was always tricky to clamp in a two-dimensional view to encircle the hepatoduodenal ligament, and it delayed the operation time for unexperienced surgeons. Bulldog clamps have been widely used in urinary surgery^[24,25]^ for vascular occlusion. In gynecology, Yang^[26]^ has expressed that the Bulldog clamp is an adequate crossover clamp with serrated blades that effectively occludes vessels without slippage or significant crush injury and is the laparoscopic instrument of choice for minimizing blood loss during surgery. However, the use of Bulldog clamps in hepatic surgery has rarely been mentioned; and this is the first report to formally demonstrate their clinical effect in hepatic surgery. In this study, we compared the use of cotton tape and Bulldog clamps for vascular occlusion during laparoscopic hepatectomy. All tourniquets were clamped successfully, regardless of the position of the patient or the presence of cirrhosis. The intermittent Pringle maneuver blocking time was consistently 15 minutes regardless of the method, with a 5-minutes release period. All the right Glisson pedicles were clamped only once to assure that the clinical data from the 2 methods were comparable. The comparison of clamping and declamping the tourniquet is indispensable. After all, we prefer laparoscopic instruments that are useful and easy to implement.

Ischemic reperfusion injury and the amount of blood loss play a significant role in liver function, which is reflected by the clamping time and the amount of blood loss. In the subgroup of operation time, the results showed that the operation time for the right segment in the Bulldog group was shorter than that in the cotton group (*P* = .003). No significant difference occurred in the left segments, which suggests the superiority of Bulldog clamps for the right segments, especially for tumors involving segments 7 and 8,^[27]^ where procedures are considered the most difficult. When we compared the clamping numbers, we found that the mean proportions of every procedure in the left segments were 1.67 and 2.1, and those in the right segments were 2.52 and 3.17, which demonstrated that ischemic reperfusion injury was worse in the traditional group (*P* = .010). Postoperative liver function was reflected by the postoperative TBIL, ALB, ALT, and AST levels. When the ALT and AST levels were compared on POD5, the Bulldog group showed significantly lower ALT and AST levels than the cotton group, which reflected the earlier recovery of postoperative liver function. The Bulldog group was associated with lower ALT and AST levels on POD1 and POD3, but no significant differences were observed between the 2 groups, which may be caused by the high proportion of right segment hepatectomies, which could also be caused by the variance in ALT and AST levels on POD1 and POD3 and the statistical variation of the small sample size. Additionally, CRP reveals a consistent response to surgical damage and evaluates the overall acute-phase reaction. Postoperative levels of CRP increase at 4 to 12 hours, peak at 24 to 72 hours and return to baseline at approximately 2 weeks.^[28]^ In our study, the level of CRP was significantly lower in the Bulldog group than in the traditional Pringle group, which demonstrates less surgical trauma in the Bulldog group; additionally, the postoperative hospital stay was lower in the Bulldog group. Among most of the data, even though most outcomes were calculated with no significant differences, intraoperative or postoperative outcomes in the Bulldog group were better than those in the cotton tape group.

### 4.1 Advantages and disadvantages

The use of cotton tape with the extracorporeal Pringle maneuver requires another incision,^[29]^ and the tourniquet location needs to be varied depending on the tumor location to easily encircle the hepatoduodenal ligament. In addition, clamping is difficult with laparoscopic instruments, which are easily entangled. Most procedures require at least once instance of blocking, so the cotton tape process in the laparoscopic context was messy for freshmen. The postoperative clinical outcomes also showed that more complications occurred in the traditional group. On the other hand, the Bulldog clamps first did not have any effect on the operation field, were easy to use, had low requirements in the laparoscopic technique, took less time and promoted the procedure. Second, small teeth covered the surface of the Bulldog clamps to tightly clamp the hepatoduodenal ligament or the right Glisson pedicle and to prevent them from moving to guarantee security. Third, the Bulldog clamps were removed without any difficulties when they were loosened to avoid prolonged ischemia reperfusion injury and were reused after disinfection. Fourth, the special material and ease of manipulation may reduce the risk of injury to the vasculature,^[30]^ bile duct and surrounding parenchyma, which contributes to the earlier recovery of postoperative liver function. Finally, the incidence of postoperative complications was lower in 54 patients with the use of Bulldog clamps.

## 5 Conclusion

The use of Bulldog clamps for vascular occlusion is effective and time saving during laparoscopic liver resection, as shown in 54 patients compared with the traditional group. The number of patients may be limited, and more information is needed to confirm the superiority of the Bulldog group, but we believe the use of Bulldog clamps will be an effective approach for suitable patients undergoing laparoscopic liver resection due to the advantages of simplicity, security and effectiveness.

## Acknowledgments

The authors would sincerely thank the reviewers and editors for critically reviewing and editing this manuscript.

## Author contributions

**Data curation:** Weixiang Li, Lei Wang.

**Formal analysis:** Weixiang Li, Hui Hou.

**Funding acquisition:** Hui Hou.

**Project administration:** Xiaoping Geng.

**Validation:** Hui Hou.

**Writing – original draft:** Liang He, Weixiang Li.

**Writing – review & editing:** Dachen Zhou, Hui Hou.
